# Comparison of nickel silicide and aluminium ohmic contact metallizations for low-temperature quantum transport measurements

**DOI:** 10.1186/1556-276X-6-538

**Published:** 2011-10-03

**Authors:** Craig M Polley, Warrick R Clarke, Michelle Y Simmons

**Affiliations:** 1 CQC2T, School of Physics, University of New South Wales, Sydney, NSW 2052, Australia

## Abstract

We examine nickel silicide as a viable ohmic contact metallization for low-temperature, low-magnetic-field transport measurements of atomic-scale devices in silicon. In particular, we compare a nickel silicide metallization with aluminium, a common ohmic contact for silicon devices. Nickel silicide can be formed at the low temperatures (<400°C) required for maintaining atomic precision placement in donor-based devices, and it avoids the complications found with aluminium contacts which become superconducting at cryogenic measurement temperatures. Importantly, we show that the use of nickel silicide as an ohmic contact at low temperatures does not affect the thermal equilibration of carriers nor contribute to hysteresis in a magnetic field.

## Introduction

Aluminium has proven to be a versatile ohmic contact metallization, and for a time was the preferred choice for silicon integrated circuits [1]. Aluminium has also been a common contact metallization for a variety of material systems such as gallium nitride [2], silicon carbide [3] and zinc oxide [4]. Owing to this versatility, aluminium has seen continued use in silicon-based research, including recent quantum dot devices for the study of quantum transport in silicon towards the goal of solid-state quantum computation [5,6].

However the characterization of such devices typically requires millikelvin temperatures, well below the normal-superconductor transition temperature of aluminium, *T*_c _= 1.175 K [7]. Below this temperature, the aluminium contacts form a Bardeen-Cooper-Schrieffer (BCS) energy gap which manifests as an increased contact resistance near *B *= 0. The contact resistance increases exponentially as the temperature is reduced, with important ramifications for studies at very low temperatures and small magnetic fields. Such studies include the measurement of electron-nuclear interactions and dephasing times [8,9], which are of critical importance for development in quantum computation [10-12]. Despite its versatility, aluminium is not an optimal metallization for low-temperature quantum transport measurements. As a result, it is important to consider alternative metallizations which do not undergo a superconducting transition at low temperatures.

In this article we examine nickel silicide (Ni*_x_*Si*_y_*) as an alternative ohmic contact metallization to silicon for use at cryogenic temperatures. NiSi has already been integrated into current CMOS processes because of its low sheet resistivity and ability to form at narrow linewidths [13]. It does not superconduct at any temperature and has recently been used in low-temperature transport measurements of a silicon nanowire quantum dot [14]. In addition, the silicide has the attractive property that it can be formed at low-temperatures, with nickel rich phases (e.g. Ni_2_Si) forming at temperatures below 350°C [15]. This property is crucial for the fabrication of atomic-precision donor-based devices where the aim is to measure transport through atomically positioned single dopants [16]. This imposes a low thermal budget to prevent diffusion of the dopants. In this article we directly compare the electrical transport properties of aluminium and nickel silicide ohmic contacts to saturation dosed *δ*-layers of phosphorus in silicon. These *δ*-layers are fabricated using identical processes to atomic-scale devices patterned by scanning-tunnelling lithography [17]. We find that nickel silicide ohmic contacts eliminate the zero-field resistance peak observed in aluminium contacts and do not introduce additional hysteresis in a magnetic field.

## Experiment

The devices were fabricated on a 1-10 Ωcm *n*-type Si(100) substrate, annealed to 1100°C in UHV by direct current heating to produce a 2 × 1 surface reconstruction. The surface was then *δ*-doped by saturation dosing with 1.1 Langmuir of PH_3 _gas at room temperature, followed by a 350°C anneal to incorporate the phosphorus into the silicon lattice [18]. After encapsulating with 30 nm of epitaxial silicon, the sample was removed from UHV to be processed into Hall bar structures. This process is known to result in 2D carrier densities of ≈ 2 × 10^14 ^cm^-2 ^[19] with dopant segregation confined to approximately 0.6 nm [20].

Electron-beam lithography and reactive ion etching were used to define the Hall bar mesas and ohmic contacts. A buffered hydrofluoric acid etch was used to remove the native oxide before the samples were loaded into a high vacuum (4 × 10^-6 ^mbar) thermal evaporator. For the aluminium Hall bars, 80 nm of Al was evaporated followed by a 30-min anneal at ≈350°C in dry N_2_. The nickel silicide Hall bars received 60 nm of Ni with a 10-nm Ti capping layer to prevent oxidation [21]. The sample was then annealed to 350°C in N_2 _for 30 min to yield the NiSi phase [15]. The unreacted nickel and titanium were removed with a sulphuric acid- hydrogen peroxide etch before Ti/Au (10/60 nm) bond pads were patterned. The Ti/Au bilayer was required for successful ultrasonic gold-ball bonding, and while bulk titanium also has a superconducting transition at approximately 400 mK [22] it is known that in thin film superconductor-normal bilayers superconductivity is strongly suppressed [23,24].

Initial magnetotransport characterization of these samples performed at 4.2 K revealed that both samples had carrier densities of (1.4 ± 0.1) × 10^14 ^cm^-2^. Subsequent millikelvin temperature measurements were performed in a dilution refrigerator that allowed simultaneous measurement of both samples with perpendicular fields up to 8 T. Magnetotransport measurements were performed using standard low-frequency lock-in techniques with a 5 nA constant current.

## Results

**Figure 1:**
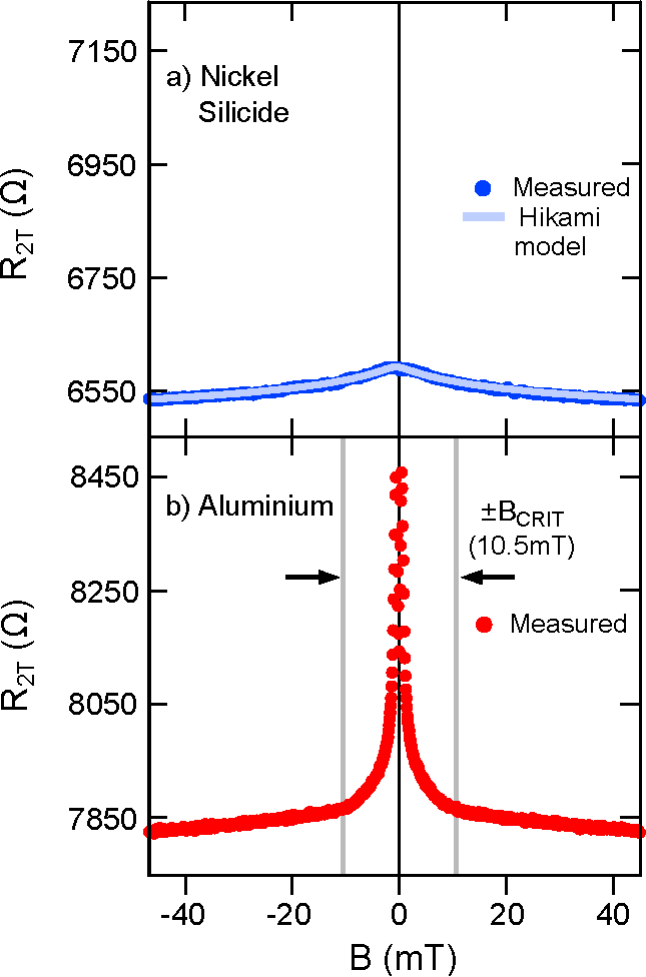
**The two-terminal magnetoresistance at base temperature (T≈50 mK) for aluminium and nickel silicide contact metallizations to the Si:P *δ*-layers**. Figure 1a shows a small peak resulting from weak localization within the *δ*-layer, and can be fitted with the Hikami model as shown. Figure 1b shows the large resistance peak around *B *= 0 that results from the formation of the BCS energy gap in the superconducting aluminium contacts. The critical field *B_C _*= 10.5 mT for aluminium is shown, which coincides with the destruction of the resistance peak.

Figure 1 compares the field-dependent two-terminal resistivities of the aluminium- and the nickel silicide-contacted Hall bars. The small resistance peak in Figure 1a originates from weak localization in the phosphorus *δ*-doped layer, where electrons become locked into phase coherent loops [25]. These loops are broken with the application of a perpendicular magnetic field, making the carriers available for transport and reducing the resistivity of the *δ*-doped layer for *B *> 0. The magnetoresistance can be well described by the Hikami model for weak localization in a disordered 2D system [26] as shown in Figure 1a, where the phase coherence length of the system (i.e. the distance electrons travel between phase randomizing scattering events) can be obtained as a fitting parameter. For the fit in Figure 1a, we obtain a phase coherence length of 450 nm, in agreement with previous studies [27]. In contrast, the magnetoresistance of the aluminium-contacted Hall bar in Figure 1b is dominated by a large peak near *B *= 0 spanning *B *= ±10.5 mT, preventing fitting to the underlying weak localization peak. This magnetic field range is consistent with the critical field *B*_C _for aluminium [7], confirming that the origin of the peak is related to the BCS superconducting gap.

**Figure 2:**
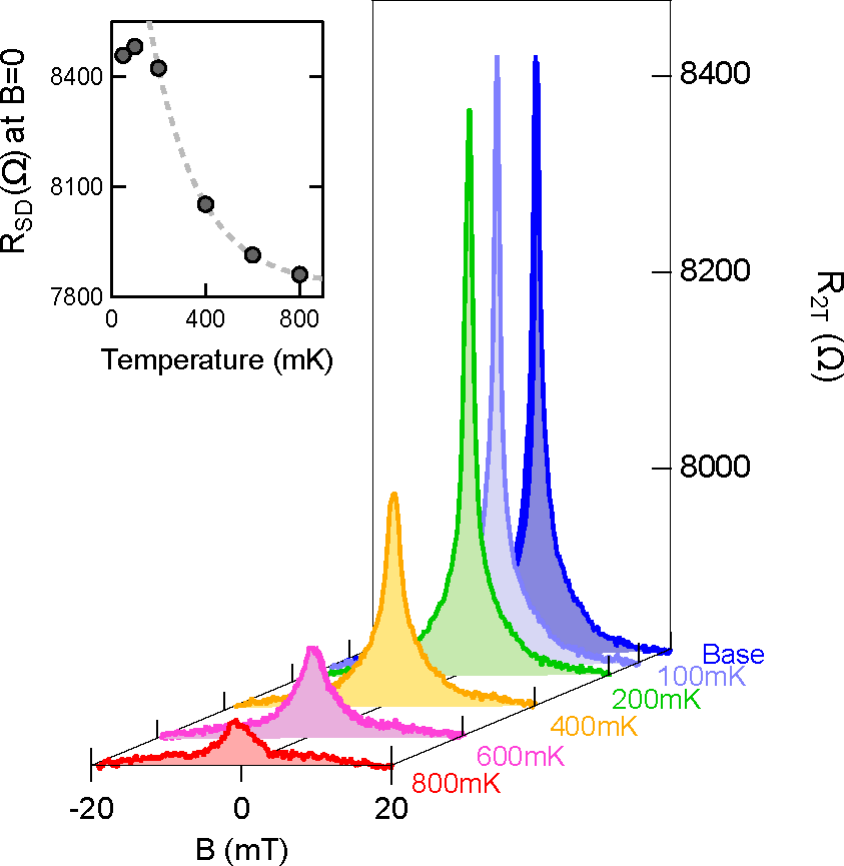
**Temperature dependence of the two-terminal magnetoresistance for the aluminium contacted Hall bar from base temperature to 800 mK**. The inset illustrates the exponential increase in the magnitude of the resistance peak, suggesting thermal activation over the BCS energy gap.

To further study the nature of this anomalous resistance peak, we have performed temperature dependence measurements as shown in Figure 2. The magnitude of the peak is seen to rapidly increase as the temperature is reduced. Whilst the BCS gap is known to increase towards a limiting value of 3.52 kT_c _as the temperature is reduced (≈ 360 *μ*eV for aluminium), it changes only weakly in the temperature range shown here (≈10%) [28]. This is therefore unlikely to cause the exponential increase in resistance shown in Figure 2. Instead we attribute this trend to the reduction of thermal energy for carrier activation over the BCS energy gap. The resistive peak continues to grow until *T *< 200 mK, at which point the electron temperature begins to saturate.

**Figure 3:**
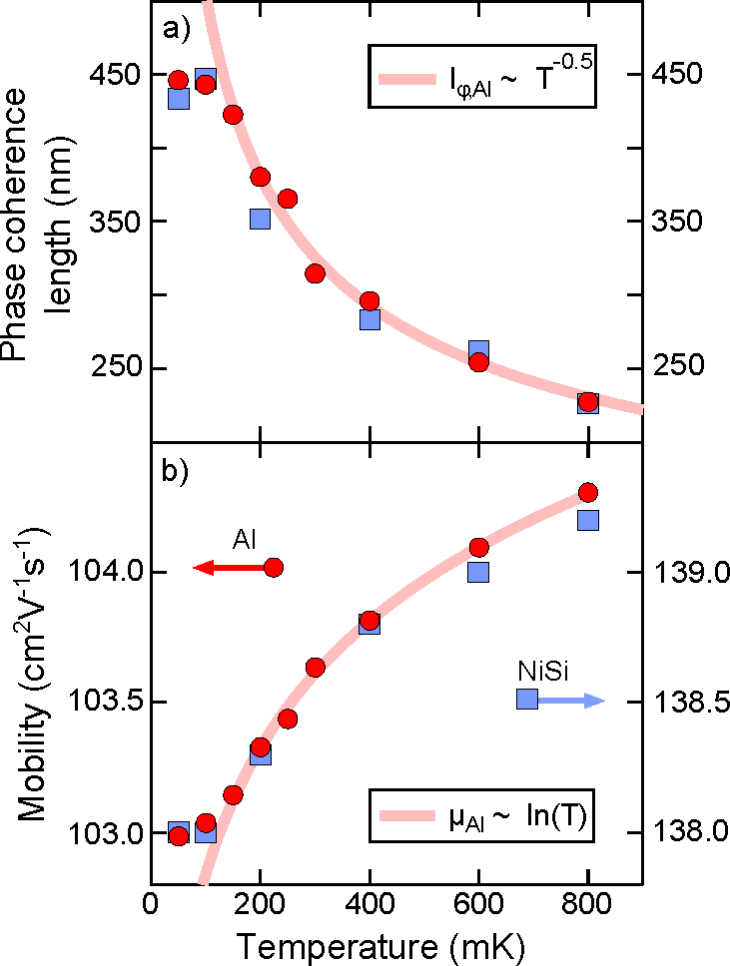
**Low-temperature magnetotransport properties of the 2D *δ*-layers as a function of temperature**. Figure 3a shows the phase coherence length as calculated from Hikami fitting while 3b shows the mobility trend. The phase coherence length is dominated by Nyquist dephasing, resulting in a *T *^-0.5 ^dependence, shown in 3a. In this regime the mobility is dominated by weak localization and electron-electron interactions, resulting in a net ln(*T*) dependence as indicated in 3b. Importantly, the temperature dependence of the mobility and phase coherence length is almost identical for both samples indicating that neither metallization is limiting the thermal equilibrium of carriers.

Both the mobility and phase coherence length can be extracted from four-terminal resistivity measurements, which eliminate contact resistance and are therefore unaffected by the two terminal resistance peaks at *B *= 0. The mobility, *μ*, is calculated directly from the measured zero-field resistivity according to the relation $\mu =\frac{1}{n_{s}e\rho }$. For highly disordered 2D systems, the phase coherence length, *l_ϕ_*, can be extracted by fitting the weak-localization peak to the Hikami model near *B *= 0, as demonstrated in Figure 1a[26]. Figure 3 shows the temperature dependence of both *μ *and the fitted values of *l_ϕ_*, and can be seen to be independent of the choice of contact metallization. The obtained values are commensurate with previous studies of *δ*-doped silicon [27]. In this temperature regime, the mobility is dominated by weak localization and electron-electron interactions, which both result in a ln(*T*) dependence [29]. Electron dephasing is dominated by Nyquist scattering, resulting in a *T *^-0.5 ^dependence for the phase coherence length [29]. The nickel silicide Hall bar has a higher mobility by ≈ 30%, which can be attributed to inhomogeneities in the initial *δ*-layer. For both samples, the mobility and phase coherence length are observed to saturate below *T *= 200 mK, confirming that the saturation of the resistive peak observed in Figure 2 is simply a consequence of the limiting electron temperature. Importantly, the fact that both samples saturate at the same temperature indicates that it is the refrigerator and not the metallization which limits thermal equilibration of carriers.

Whilst pure nickel is ferromagnetic, previous theoretical study has concluded that transition metal silicides including NiSi are diamagnetic [30]. However previous experimental results have indicated ambiguity in the magnetic properties of NiSi for fields below 200 mT at low temperatures [31]. It is therefore important to determine whether the nickel silicide contacts used here have any influence on the measured magnetic field hysteresis.

**Figure 4:**
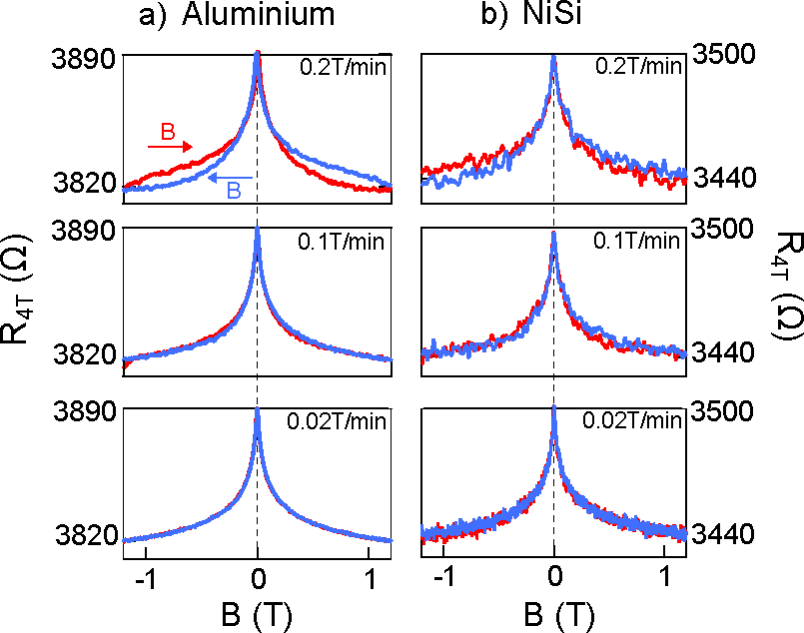
**Dynamic hysteresis in the magnetoresistance measured at base temperature**. Figure 4a shows the hysteresis in the magnetoresistance of the aluminium contacted Hall bar as a function of magnetic field sweep rate. At a fast sweep rate of 0.2 T/min clear hysteresis is observable, but disappears for sweep rates of 0.1 T/min or lower. Figure 4b shows the same results from a nickel silicide contacted Hall bar. We see comparable behaviour, indicating that the nickel silicidation process does not exacerbate the hysteresis.

We have measured the four-terminal magnetoresistance for both metallizations as a function of magnetic field for different magnetic field sweep rates as shown in Figure 4. Particular care was taken to ensure that the magnetic environment of each sample was identical. To this end, the samples were measured sequentially (several days apart) using the same package in the same dilution refrigerator configuration. Magnetic hysteresis is seen for both samples with fast sweep rates of 0.2 T/min, cooling the sample as the field sweeps towards *B *= 0 and heating as the field sweeps away from *B *= 0. This is characteristic of adiabatic demagnetization of a ferromagnetic material, where thermal and magnetic energies are exchanged faster than the cryostat can equilibrate. Figure 4 shows that the level of hysteresis is similar in both samples, suggesting that it is the ferromagnetic impurities in the immediate environment rather than the ohmic contacts that are responsible for this effect. For both samples, the hysteresis can be eliminated by decreasing the magnetic field sweep rate to < 0.1 T/min to allow sufficient time for the system to equilibrate. We note that the slight difference in noise between Figure 4a,b is because of the different measurement electronics used for the second series of measurements. Within each measurement set the noise levels were comparable between the samples.

## Conclusions

We have compared the low-temperature magnetotransport properties of highly doped Si:P *δ*-layers with both nickel silicide and aluminium ohmic contacts. We have shown that a nickel silicide contact is comparable to aluminium, with the added advantage that nickel silicide does not transition to a superconducting state at low-temperatures (*T *< 200 mK). This eliminates the contact resistance peak around *B *= 0 observed with superconducting aluminium contacts, important for measurements of electron-nuclear interactions and de-phasing times. In addition, we have shown that nickel silicide contacts neither alter the thermal equilibration of carriers nor contribute to hysteresis in a varying magnetic field.

## Competing interests

The authors declare that they have no competing interests.

## Authors' contributions

CMP fabricated and measured the samples and wrote the manuscript. WRC and MYS assisted in experimental design, measurement, data analysis and preparing the manuscript.
